# Indices of Increased Decompression Stress Following Long-Term Bed Rest

**DOI:** 10.3389/fphys.2018.00442

**Published:** 2018-07-18

**Authors:** Mikael Gennser, S. L. Blogg, Ola Eiken, Igor B. Mekjavic

**Affiliations:** ^1^Swedish Aerospace Physiology Centre, Department of Environmental Physiology, CBH, KTH Royal Institute of Technology, Stockholm, Sweden; ^2^SLB Consulting, Cumbria, United Kingdom; ^3^Department of Automation, Biocybernetics and Robotics, Jožef Stefan Institute, Ljubljana, Slovenia; ^4^Department of Biomedical Physiology and Kinesiology, Simon Fraser University, Burnaby, BC, Canada

**Keywords:** bed rest, extravehicular activity, decompression sickness, venous gas emboli, space simulation

## Abstract

Human extravehicular activity (EVA) is essential to space exploration and involves risk of decompression sickness (DCS). On Earth, the effect of microgravity on physiological systems is simulated in an experimental model where subjects are confined to a 6° head-down bed rest (HDBR). This model was used to investigate various resting and exercise regimen on the formation of venous gas emboli (VGE), an indicator of decompression stress, post-hyperbaric exposure. Eight healthy male subjects participating in a bed rest regimen also took part in this study, which incorporated five different hyperbaric exposure (HE) interventions made before, during and after the HDBR. Interventions i–iv were all made with the subjects lying in 6° HD position. They included (C1) resting control, (C2) knee-bend exercise immediately prior to HE, (T1) HE during the fifth week of the 35-day HDBR period, (C3) supine cycling exercise during the HE. In intervention (C4), subjects remained upright and ambulatory. The HE protocol followed the Royal Navy Table 11 with 100 min spent at 18 m (280 kPa), with decompression stops at 6 m for 5 min, and at 3 m for 15 min. Post-HE, regular precordial Doppler audio measurements were made to evaluate any VGE produced post-dive. VGE were graded according to the Kisman Masurel scale. The number of bubbles produced was low in comparison to previous studies using this profile [Kisman integrated severity score (KISS) ranging from 0–1], and may be because subjects were young, and lay supine during both the HE and the 2 h measurement period post-HE for interventions i–iv. However, the HE during the end of HDBR produced significantly higher maximum bubble grades and KISS score than the supine control conditions (*p* < 0.01). In contrast to the protective effect of pre-dive exercise on bubble production, a prolonged period of bed rest prior to a HE appears to promote the formation of post-decompression VGE. This is in contrast to the absence of DCS observed during EVA. Whether this is due to a difference between hypo- and hyperbaric decompression stress, or that the HDBR model is a not a good model for decompression sensitivity during microgravity conditions will have to be elucidated in future studies.

## Introduction

Human extravehicular activity (EVA) is an essential part of space exploration, having taken place on the moon, and continuing from space vehicles and the International Space Station (ISS). The hostile environment of space means that protective suits have to be worn during EVA to guard against extreme temperatures, radiation and effectively, no atmospheric pressure. Although the ambient pressure in the ISS is the same as that at sea level on earth (1 ata or 101 kPa), the pressure maintained inside the space suit is significantly lower, to allow some flexibility of movement. The Russian ‘Orlan’ suit has an internal pressure of 38.6 kPa (comparable to 7440 m altitude), while the United States extravehicular mobility unit (EMU) suit maintains 29.6 kPa (9250 m) (Norfleet and Butler, 2001). This means that personnel are subject to decompression when moving from ISS ambient pressure to that of the suit.

Concomitant with decompression is the risk of decompression sickness (DCS). A number of studies have examined DCS risk at altitudes comparable to the corresponding internal pressure of the space suits mentioned above. Overall, the United States Space program found a 20–40% DCS incidence with ground-based simulated EVAs (Conkin, 2001), although these protocols have produced no reported incidence of DCS in space (Conkin et al., 2016).

It has been hypothesized that microgravity might be protective against both the growth of venous gas emboli (VGE) and incidence of DCS. Reasons for this hypothesis include the positive effect of weightlessness, and adynamia (loss of strength). Balldin et al. (2002) showed that simulated weightlessness (supine position) did not provide any protection against DCS incidence over a control ambulatory group after both groups were exposed to hypobaric conditions, even though there was a significantly higher incidence of bubbles in the ambulatory subjects. Conkin et al. (2017) found a higher incidence of VGE and DCS in subjects who exercised before and during hypobaric exposure than in those who did not exercise during the hypobaric exposure, despite both groups undertaking a prebreathe with exercise to denitrogenate themselves. Another study (Conkin and Powell, 2001) compared lower body adynamia (LBA) that included upper body exercise while at altitude with random walking exercise and planned exercise. They found that LBA treatment appeared to protect against DCS and VGE as effectively as random walking and more than following planned exercise.

The nearest we can come to simulating the effects of microgravity on earth is with a 6° head down tilt bed rest (HDBR) experimental model, which causes a pooling of fluids in the upper body similar to that seen in microgravity (Charles and Lathers, 1991). In the longer term, it can also simulate the wasting seen in the musculoskeletal system after a period spent in space. In the present study, an extended period of bed rest (5 weeks) was used to investigate various resting and exercise regimen on the formation of VGE post-hyperbaric exposure.

In hypobaric exposures, decompression is not preceded by a period of increased pressure as in diving but is initiated immediately from saturation at ambient pressure. In a study where exercise (150 knee bend squats) was taken immediately before hypobaric decompression to 6706 m, there was a significant increase in VGE production in comparison to the numbers formed when the squats were performed 1 or 2 h prior to the decompression (Dervay et al., 2002). This led the authors to hypothesize that the micronuclei formed by the eccentric exercise had a half-life of around 1 h under the conditions of the study.

Conventionally, exercise at depth is thought to raise the risk of DCS (Francis and Mitchell, 2003), however, in one study no differences in post-dive VGE were found between exercise at depth and no exercise (Jankowski et al., 2004). Exercise during decompression may also have an effect on inert-gas saturation and the amount of VGE produced post-dive. Studies have found that moderate exercise performed during decompression can reduce VGE production and the risk of DCS (Jankowski et al., 1997, 2004). Conversely, cramped body positions in the decompression phase may reduce inert gas washout and so increase DCS risk (Guilliod et al., 1996). In this regard, hypobaric exposures may produce different results; Balldin et al. (2002) found that a group walking around in a chamber during a 4 h hypobaric exposure to 29.6 kPa produced more VGE at altitude than subjects performing occasional mild exercises while maintaining a supine position for the duration of the exposure. However, the incidence of DCS did not differ between the groups. Karlsson et al. (2009) also found that supine inactivity reduced the formation of VGE during acute altitude exposures.

Post-hyperbaric or hypobaric exposure, there is some evidence that exercise, both isometric and isotonic, made in the peri-decompression period may increase the risk of DCS occurring (Pollard et al., 1995). Pilmanis et al. (1999) could find no difference (40% occurrence) between the two modes of exercise in eliciting DCS post-hypobaric exposure to 8992 m for 4 h.

The aim of the present study was to investigate whether prolonged bed rest would have an effect on the amount of VGE produced post-hyperbaric exposure, in comparison to a condition of short supine rest prior to the pressure exposure. Also, hyperbaric exposure (HE) after prolonged bed rest was compared with exercise taken immediately before, during and after the HE. We tested the null hypothesis that the bed rest treatment would give rise to less VGE production than the control situations.

## Materials and Methods

### Subjects

Ten healthy male subjects gave their written informed consent to participate in the study. Their body mass ranged from 63 to 98 kg (mean ± SD: 75 kg ± 10 kg), their body mass index (BMI) from 21.0 to 27.2 (mean ± SD: 23.4 ± 2.1) and their age from 21 to 28 years (mean ± SD: 23 ± 2) (**Table 1**).

**Table 1:** Subjects’ physical characteristics.

Subject no.	Age (years and months)	Height (cm)	Weight (kg)	BMI	Weight change C1–C4 (kg)
1	22.5	191.3	80	21.0	-5.8
2	26.0	176.3	75	23.9	+0.4
3	20.8	175.5	82	26.9	-2.8
4	22.4	176.2	71.5	22.9	-3.1
5	22.1	181.5	76.5	21.7	-2.9
6	22.9	177.0	75	22.9	-3.0
7	28.1	171.5	66	22.0	-2.8
8	23.4	172.5	69	23.0	+1.1
9^∗^	23.4	176.2	72	22.7	+0.3
10^∗^	21.8	191.0	100	27.2	-11.2


*^∗^Not included in statistical analysis.*

All were participating principally in a long term (35 days) 6° head down bed rest study (HDBR) at the Valdoltra Orthopaedic Hospital (Ankaran, Slovenia). Subjects gave their informed consent to participate in all parts of the study and were free to leave the trial at any time. The study protocol and experimental procedures were in accordance with the Declaration of Helsinki and were approved by the Committee for Medical Ethics at the Ministry of Health of the Republic of Slovenia. For practical reasons, only 10 subjects could be included in the bed-rest regimen. In addition to the 6° HDBR, the subjects also consented to take part in the present study, which required that they were subjected to a number of HEs, before, during and after the bed rest. The exposures involved a variety of interventions, with dives made in a dry hyperbaric chamber at the Josef Stefan Institute, Ljubljana, Slovenia.

### General Bed Rest Procedures

The duration of the HDBR was 35 days. Subjects were accommodated in two rooms and remained in the 6° head-down position at all times. Subjects were allowed one pillow and could occasionally lean on one elbow to eat or while being moved to a stretcher. Their arms were allowed to move in and above the horizontal plane, but legs were kept in the tilted plane at all times. Muscular exercise was prohibited. Although they were not allowed alcohol, other food and drink was not restricted and subjects were provided three nutritionist-compiled meals per day.

To ensure that the subjects complied with the study regimen and restrictions at all times, and also for subject safety, video cameras provided 24 h surveillance. Each subject received physiotherapy twice a week and also on request, in order to mitigate neck/back pain and stiffness of joints due to bed rest. The therapy was performed in the HDBR position and consisted of massage, assisted (passive) stretching and assisted joint flexion. Each subject was screened for deep vein thrombosis twice a week, using an Ultrasound/Doppler system with a 6.0–11.0 MHz linear array transducer (Aspen, Acuson, Mountain View, CA, United States) to visualize the popliteal veins bilaterally.

Following bed rest, each subject resumed their normal ambulatory lifestyle and also participated in a supervised exercise training program, consisting of 11–12 1 h sessions of cycle ergometry or lower body resistance training. (For further information regarding the bed rest procedure see Eiken et al., 2008.)

### Hyperbaric Exposures

Subjects were split into pairs for their HE. Each HE followed the United Kingdom Royal Navy standard air Table 11, with 100 min spent at 18 m (280 kPa). Decompression commenced at a rate of 15 m/min, with a stop at 6 m for 5 min, and another at 3 m for 15 min. The floor of the hyperbaric chamber was adjusted to tilt to the same angle (6^°^) as that of the beds in the HDBR study, with the subjects remaining in a head-down, supine position during each pressure exposure. For the HE that was made during the HDBR period (see T1 below) subjects were transported, lying in a 6° HD position, to the Josef Stefan Institute in an ambulance.

### Hyperbaric Protocols

**FIGURE 1:**
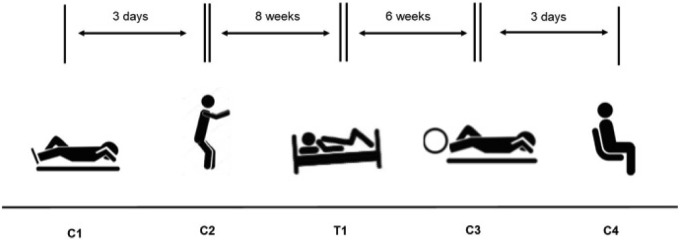
Experimental schedule for the four control hyperbaric exposures (C1–C4) and the test hyperbaric exposure (T1).

Initially the study was designed to compare bubble data collected after the control HE (C1 – see below, **Figure 1**) against that collected after the HE made during the HDBR period (T1). However, after C1, the subjects produced very low numbers or no Doppler detectable bubbles, so further controls (C2–C4) were brought into the study, each adding a facet to the investigation (exercise prior to the HE – C2; exercise during the HE – C3; subjects upright and ambulatory – C4). This was necessary, as if no bubbles could be detected then there would be nothing to provide a comparison for the bubble grades measured during the HDBR, given that our initial hypothesis was that the HDBR would give rise to less VGE than the control situation.

Control 1 (C1) – Performed prior to the HDBR study, to provide baseline control data. The subjects lay down outside the chamber at the Josef Stefan Institute for 1 h prior to the HE. They were carried inside chamber while supine, were subjected to the dive, then lifted out and remained supine for 2 h while post-decompression Doppler measurements were made.
Control 2 (C2) – Also performed prior to the commencement of the HDBR study. Subjects lay at rest for 1 h prior to the dive, but performed 150 knee bends immediately before entering the chamber over a period of 10 min. The subjects then walked inside and lay down during the HE. Following the HE, the subjects were carried out of the chamber and remained supine while the post-decompression measurements were made over a 2 h period.
Treatment 1 (T1) – Performed during the last week of the HDBR study. The subjects were transported to and from the Valdoltra Orthopaedic Hospital and the Josef Stefan Institute by ambulance, lying in the HDBR position on the stretchers at all times. They remained supine while carried into the chamber, during the HE and during the 2 h of post-dive measurements. They were then transported back to the Valdoltra hospital to finish their bed-rest period.
Control 3 (C3) – Performed 6 weeks after the end of bed rest. Again, subjects lay down for 1 h prior to the HE at rest, were then carried into the chamber and remained supine during the HE. They performed some light cycling on their backs while at depth (Monark cycle ergometer; Idass, Glastonbury, United Kingdom), working at 50 W for 10 min, then resting for 10 min and so on throughout the 100 min bottom time. The subjects were carried from the chamber and again remained supine for 2 h during their post-decompression measurements.
Control 4 (C4) – This treatment was also performed 6 weeks after the completion of the bed rest. The subjects lay at rest for 1 h prior to entering the chamber. They were then allowed to walk into the chamber and sat upright during the HE, then walked out at the end of the HE. During the 2 h post-decompression measurement period, subjects were allowed to move around in between the measurements. Doppler measurements were made on supine subjects, but for flex, each subject stood up and did three knee bends, then lay again for measurement to be graded.

C3 and C4 were performed in the same week. On the first dive, one subject in each pair cycled in the chamber, while one sat upright. Three days later, the pairs returned with the one who had cycled now sitting upright and the other cycling.

In all cases, if necessary, the subjects were covered by blankets when lying outside chamber to keep them warm.

### Doppler Protocol

Precordial Doppler audio measurements were made using a Doppler Bubble Monitor (DBM9008; Techno Scientific Inc., Ontario, Canada) to evaluate any VGE produced post-decompression. VGE were evaluated on the Kisman Masurel scale (Kisman et al., 1978) by an experienced operator, with measurements made at 5 min intervals for the first 30 min after decompression, then at 15 min intervals for a total period of 2 h. Measurements were made while the subjects were at rest in the left lateral decubitus position and also after a ‘flex’, which involved the subjects kicking their feet away from their body vigorously three times then coming back to rest. This protocol was followed for all of the treatment modes bar number five, where the flex measurements were made after the subject stood up and made three knee bends, then lay down again. As Doppler grades are ordinal data, they are represented by Roman numerals in the text (III, IV etc.).

### Statistics

Maximum bubble grades were noted and the Kisman integrated severity scores (KISS) (Jankowski et al., 1997) were calculated for each subject following each treatment. Although an indirect relationship, the higher the bubble load, the more likely DCS is to occur (Francis and Mitchell, 2003), and large numbers of bubbles over a protracted period indicate a high free-gas load so increasing the risk of clinical symptoms (Spencer and Johanson, 1974; Yount and Hoffman, 1986). Therefore, maximum grades are useful to illustrate the highest number of VGE and to infer some idea of DCS risk to the individual at a particular point in time. In addition, the KISS method denotes an ‘index of severity’ for each protocol, integrating all of the detected bubbles over the measurement period for each subject. KM Doppler grades are ordinal data, therefore statistical testing is usually performed using non-parametric tests such as the Friedman test and Wilcoxon signed rank test. However, in this experiment where several control situations were added, the low power of these tests would make it difficult to discern any differences. Also, there are no generally accepted *post hoc* tests for these non-parametric tests. Therefore, as suggested by Baguley (2012) the data for the maximum Doppler scores or KISS scores were rank transformed and then a one-way ANOVA for correlated samples performed on the ranks. The differences between pairs of treatments were tested using Tukey HSD test. The data was calculated on the VassarStats website for statistical computation. Comparisons (ANOVA) were made between the supine controls (C1–C3) and the HDBR situation (T1), and between the supine and the upright control (C4). The significance level was set at *p* < 0.05.

## Results

Although ten subjects entered the study, the results of eight subjects only were used in the analysis. Of the two excluded, one (subject BB – see **Table 1**) had already experienced substantial weight loss (≈ 20 kg) before being enlisted in the study and this continued during HDBR [change in weight from first measurement (C1) to last (C4) was 11.2 kg; see **Table 1**]; it was felt that the VGE data might be affected by this weight loss and change in BMI. The change in BMI for this subject, –2.9 kg/m^2^, was larger than the average for the remaining nine subjects plus 3.7 standard deviations. The second subject (SB) was excluded as none of the hyperbaric treatment protocols elicited any VGE in him.

Following C1, only one subject (subject three) produced VGE in the 2 h post-dive measurement period, producing a maximum grade of KM III, with a relatively high KISS score of 34.3 indicating a high bubble load. Across all subjects, the median KISS score for C1 was zero. Following C2, where the subjects performed deep knee bends immediately prior to entering the HE, two subjects (two and six) produced bubbles post-decompression and once again the median KISS was zero. All subjects apart from one (subject seven) produced bubbles after T1 (during the last week of the HDBR). Five of the subjects produced a maximum KM Grade of II or above and the median KISS score was 0.8. After C3, which involved supine cycling at depth, only two subjects produced a small number of VGE (subjects two and three; maximum KM I), with a median KISS of zero. After C4, where the subjects were allowed to sit upright during the HE and move around between measurements post-decompression, VGE were produced in five subjects, with maximum KM grades ranging from I–III and a median KISS of 1, the highest across all of the treatments.

**FIGURE 2:**
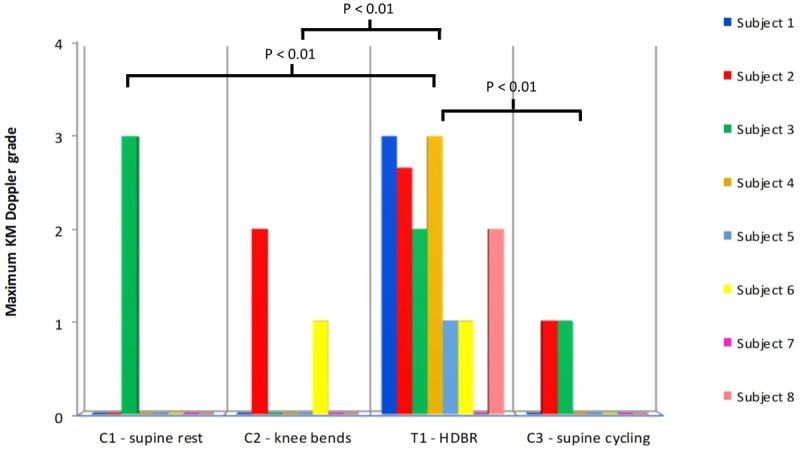
The maximum KM Doppler grades for each subject after all of the supine controls (C1–C3) plus the HDBR treatment (T1). Statistical testing was performed on ranked data (ANOVA: *F* = 7.64, *p* = 0.00123). Only statistically significant pair-wise differences are indicated in the figure. No signs or symptoms of DCS were noted at any point.

Statistical comparison (ANOVA) of the ranked maximum VGE data for the supine control groups (C1–C3) vs. the HDBR (T1) (see **Figure 2**) revealed a significant difference between the groups (*p* = 0.00123). There was a difference between all the controls and T1 (*p* < 0.01), but none of the control situations (C1–C3) differed between each other indicating that the HDBR produced significantly higher maximum bubble grades than the supine control treatments, most of which involved exercise before or during the HE (C2 and C3). Similar results were shown for the KISS scores. Statistical comparison (ANOVA) of the ranked KISS scores for the supine control groups (C1–C3) vs. the HDBR (T1) showed a significant difference between the groups (*p* = 0.00174). There was a difference between all the controls and T1 (*p* < 0.01), but none of the control situations (C1–C3) differed between each other indicating that the HDBR condition produced significantly more bubbles than the supine control treatments. In short, both bubble indices showed that overall, the number of VGE produced by a decompression challenge post-HDBR was larger than after the supine control situations.

**FIGURE 3:**
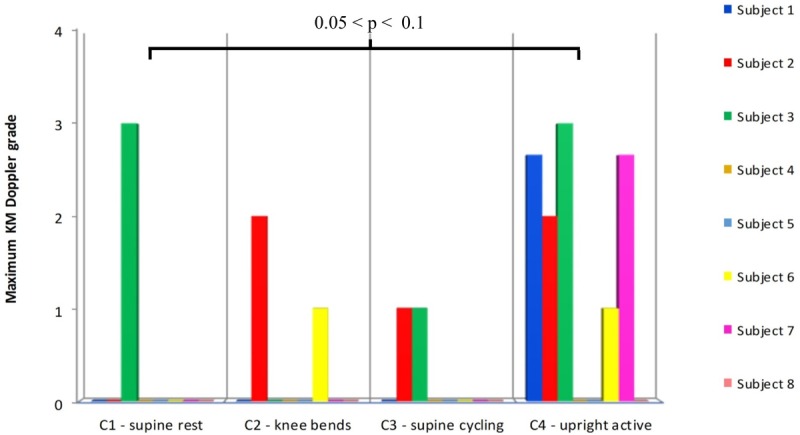
Comparison of the maximum Doppler grades produced after the supine controls (C1–C3), with the control (C4) measurements made 6 weeks after the end of the HDBR, where the subjects were allowed to sit upright in the chamber and move around after HE. Statistical testing was performed on ranked data (ANOVA: *F* = 3.18, *p* = 0.045). Difference between C1 and C4 *p* = 0.061 (Tukey’s HSD).

Comparison of the ranked maximum KM grades for C1–C4 showed a significant (between groups) effect (ANOVA, *F* = 3.18, *p* = 0.046) (**Figure 3**). However, none of the individual comparisons were statistically significant (C1 vs. C4 *p* = 0.061), although the KM scores for the upright/ambulant were nominally the largest. The KISS results did not differ significantly between these groups.

## Discussion

The present study shows that a 5 week HDBR increases decompression stress after a HE. During space explorations, upon decompression from normal atmospheric pressure to a hypobaric pressure, astronauts are subject to decompression stress. Unfortunately, it was not technically possible to achieve hypobaric decompressions in this study, as a hypobaric chamber was not available in the vicinity. The difference between a hypobaric decompression from normal atmospheric pressure (in essence, a saturation decompression) and a hyperbaric decompression is twofold; firstly, tissue compartments with longer half-times are stressed during the hypobaric decompression, and secondly, the hyperbaric decompression entails two phases, the wash-in phase when the tissue partial pressure of dissolved gas is increased, and the decompression phase.

The fact that the slower compartments were not challenged in this study is a difference that will have to be borne in mind when comparing these results with decompressions experienced during space walks. The difference in the gas wash-in phase is unlikely to have been of any major importance given that the control test, where the subjects exercised lightly in a supine position during the HE, was not different from the other resting supine control tests (C3 vs. C1, C2).

As shown in **Figure 2**, the maximum bubble grades were significantly lower in the supine controls (C1–C3) than after the HDBR (T1). There was no significant difference between the supine control (C1) maximum bubble grades and the upright control (C4), although there was a tendency toward higher grades in the latter (**Figure 3**). It should be noted that the bubble grades observed following the supine control treatments (C1–C3) were surprisingly low. The dive profile chosen for the study (United Kingdom Royal Navy Table 11) has been used to provoke bubbles in a number of trials investigating prophylactic measures to guard against DCS (Blogg et al., 2010, 2017; Jurd et al., 2011; Gennser et al., 2012), as it is known to regularly produce VGE loads across the complete range of the KM grading scale, but with a low incidence of DCS. However, in the present study, hardly any bubbles were produced following the control HE. Although some subjects’ maximum grades were toward the high end of the scale on occasion (**Figure 2**), KISS scores, which give an impression of overall bubble load, were quite low across all of the treatments.

The reason for this scarcity of bubbles is not known but could be explained to some extent by the fact that the subjects in the present study were all young and fit (mean age 23 and mean BMI 23.4), which is in contrast to other studies. For example in the study by Gennser et al. (2012), the mean age of the subjects was 40 years, with a mean BMI of 27.7. Further, Conkin et al. (2003) found that age was significantly related to VGE load, with younger subjects having fewer bubbles. In addition to their young age, the subjects were supine during the whole HE and during the post-exposure measurement period. Although Balldin et al. (2002) did not find any protective effect of supine rest on altitude DCS, they did find a significantly lower incidence of bubbles in supine subjects (81% vs. 51%). Similarly, Karlsson et al. (2009) noted very low bubble grades in subjects exposed to high altitude during supine rest. Whatever the reason for the very low amount of bubbles produced by the subjects after the supine control dives, this low level of bubbling makes the difference between it and the significantly higher maximum grades produced after the HDBR (T1) more obvious.

There are two potential reasons why HDBR may produce more bubbles post-hyperbaric exposure. The first is concerned with peripheral vasoconstriction provoked during HDBR. In a study investigating core temperature during 35 days of bed rest, it was found that skin temperature decreased progressively over the period, with the distal regions being affected the most (Golja et al., 2002). An associated reduction in blood flow to the peripheral areas would reduce wash-out of inert gas upon decompression, so supersaturation of these tissues and bubble production therein would be more likely post-decompression. This is in direct contrast to the positive effect of weightlessness encountered in space walks, which increases peripheral blood flow back to the heart (Arborelius et al., 1972). (Obviously, the fact that a reduced peripheral blood flow may also have been present during the period at pressure would reduce the wash-in rate of nitrogen. However, given the rather long period at pressure, the reduced wash-in rate would only affect the tissues with longer half-times.)

However, if peripheral vasoconstriction was responsible for the high bubble production seen after the bed rest, then likewise exercise at depth should produce an increased number of bubbles. The period of exercise would serve to increase blood flow, metabolism and inert on-gassing to all areas of the body during the at-depth period, so causing a greater net inert gas balance with the potential to form more VGE post-decompression. Yet it was found that on comparison with the HDBR (T1) data, the maximum bubble grades observed after exercise at depth (C3) were significantly lower (**Figure 2**). Also, as has been mentioned previously, there was no difference between the supine control pressure exposure with exercise during the hyperbaric phase (C3) and the other supine control pressure exposures (**Figure 3**).

The second possible explanation for these results is that with the extended period of rest afforded by the HDBR, bubble micronuclei located in the endothelial walls of the blood vessels were not destroyed as they might be, if high impact exercise had been undertaken. It is assumed that bubble micronuclei form naturally and would continue to do so during bed rest. Exercise is thought to be able to both create nuclei through injury and cavitation (Vann and Thalmann, 1993; Dervay et al., 2002) and to destroy them (Germonpre et al., 2009; Jurd et al., 2011), with the latter studies suggesting that the net balance is toward the reduction of subsequently formed decompression bubbles, so long as the appropriate form of exercise is undertaken.

Historically, exercise before HE (e.g., diving), particularly that strenuous enough to cause muscle soreness, was thought to be a risk factor for DCS. Microscopic muscle tears were considered sites where micronuclei, and later larger bubbles, could form and grow (Vann and Thalmann, 1993). However, recent studies show that certain types of exercise prior to diving and decompression are protective in terms of reducing VGE formed post-dive, though the timing and the mode of any beneficial exercise has proved to be contentious. Initial work indicated that in rats, a bout of high-intensity exercise performed on a treadmill 10–20 h before a dive, but not thereafter, reduced VGE formation post-dive (Wisloff et al., 2004). Dujic et al. (2004) corroborated these positive findings in a similar study using human subjects performing 280 kPa dry chamber dives, where treadmill running 24 h prior to the dives also reduced post-dive VGE in comparison to no exercise. Further studies investigated the effect of exercise taken closer to the dive time. Recent work in humans involving both medium and high intensity running exercise commencing 2 h prior to a dive, was found to reduce VGE formation (Blatteau et al., 2005, 2007). Similarly, medium or high-intensity cycling exercise commencing 2 h before an open water dive also reduced VGE grades (Pontier and Blatteau, 2007). Endurance running (45 min continuous sub-maximal) exercise immediately before diving was also shown to significantly reduce VGE formation in comparison to control (Castagna et al., 2011). However, sub-maximal cycling at either 24 or 2 h prior to a dive was not shown to be beneficial in terms of reduction of VGE in another study (Gennser et al., 2012).

These contrasting data indicate that a complex relationship exists between exercise and VGE production. The mechanisms involved may include nitric oxide production, haemodynamics and fluid balance, as well as the mode of exercise undertaken and its effect on the formation of bubble micronuclei. Thus, when the sub-maximal exercise study (Gennser et al., 2012) was repeated, using sub-maximal running/jumping exercise instead of cycling, it was found that replacing the concentric exercise with moderate-intensity impact exercise 2 h prior to a dive caused VGE formation to be significantly reduced post-dive (Jurd et al., 2011). This suggests that high impact exercise might be capable of dislodging gas nuclei in the blood vessels, a hypothesis that was supported further by a study investigating 30 min of whole-body vibration made 1 h before a dive (Germonpre et al., 2009), as VGE formation was again significantly reduced in comparison to non-vibrated control.

In contrast to the positive effect of exercise, inactivity has been shown to have a deleterious effect on the vascular system and among other consequences cause an increase of endothelial microparticles in the blood (Navasiolava et al., 2010; Boyle et al., 2013). Microparticles are small vesicles released from active and injured endothelial cells (Thom et al., 2013). It has been shown that these particles are compressible by an applied pressure of 790 kPa, and pre-pressurization to that pressure abolished the gas phase (Thom et al., 2013). Here it is relevant to consider the second control experiment when subjects were asked to perform squats prior to the compression. A similar experiment prior to hypobaric exposure has shown an increase in circulating bubbles when the squats were performed just prior to the decompression (Dervay et al., 2002). It was estimated that the bubble nuclei apparently created by the squats had a half-time of approximately 1 h. In the present study, the squats were performed just prior to the start of compression, but 100 min prior to the start of decompression. Thus, the finding that the squats did not increase the amount of bubbles post-decompression may be explained partly by the time delay to the decompression (the chamber did not allow the subjects to stand upright and perform squats) and partly by the pressure increase that would act to compress any pre-existing gas phase. Therefore, for microparticles to be able to act as bubble nuclei during the subsequent decompression they must either be able to withstand the pressure, or form a gas-phase during the period at pressure prior to decompression. It might be hypothesized that gas nuclei formed during prolonged bed rest have more time to stabilize than microbubbles produced during a short bout of exercise.

This idea has gained some support by recent observations that hydrophobic spots on the luminal surface of blood vessels serve to promote stable nanobubbles, which when exposed to gas supersaturation form decompression bubbles. When isolated vessels were exposed to mechanical stresses *in vitr*o, bubbles were released. The released bubbles appeared to deplete the vessel wall of the hydrophobic material, and thus reduce the subsequent propensity for bubble formation (Arieli et al., 2015).

If one accepts that vascular bubbles are indicative of decompression stress and are related to the risk of DCS (Spencer and Johanson, 1974), then it would appear that bed rest is not a good simulation of microgravity for decompression risk. In space flight, astronauts would be active for most of their waking hours, so they would likely be creating and destroying micronuclei constantly. Although it is known that long space flights induce changes in the vasculature (Charles and Lathers, 1991), it is not known whether excessive microparticle production takes place. There were no cases of DCS in the present study, and overall the bubble loads observed in the young, healthy subjects were relatively low, but it was the HDBR treatment that provoked the largest maximum bubble grades. Although HDBR causes a pooling of fluids in the upper body and the wasting of the musculoskeletal system similar to that seen after a period spent in space, it would seem that it does not simulate the potentially positive benefits of mobility in microgravity that might help to balance the equation and reduce the risk of DCS, leading to the low incidence of DCS reported in astronauts.

The only control situation that came close to be as bubble producing as the HDBR situation was the upright control (C4), where the subjects were allowed to sit up inside the chamber and move around during the Doppler measurement period. Although the subjects were not exercising during the post-decompression monitoring period *per se*, during the upright control treatment, they performed standing knee bends for the flex Doppler measurement, which is a fairly strenuous muscular activity. It is generally accepted that post-dive exercise increases DCS risk, but there are few studies on this topic. Pollard et al. (1995) determined in rats that post-dive exercise (30 min walking) produced a significantly greater occurrence of DCS than did rest after diving. Van Der Aue et al. (1949) exercised human subjects with arm and leg weight lifting after a variety of long no-stop dives that pushed the boundaries of modern dive profile conservatism. The exercise elicited a 47% occurrence of DCS in comparison to a 22% incidence in resting controls.

Neither the level nor the type of activity performed by a diver or subject post-decompression is often considered closely or well described when monitoring post-decompression bubbles and subsequently managing DCS risk. However, the observation of increased amount of bubbles in the upright control situation compared to the supine controls, despite falling short of a statistical significant difference, indicates the need for a close control of the activity during the post-decompression monitoring period in comparative studies of decompression stress.

## Conclusion

In contrast to the suggested protective effect of pre-dive exercise on bubble production, a prolonged period of bed rest prior to a HE appears to promote bubbling post-decompression. HDBR does not seem to be a good model with regards to decompression stress in microgravity when the decompression stress is via HE. Whether long-term bed rest has a different effect on hypo- and hyperbaric decompression stress, will have to be clarified in future studies.

## Author Contributions

MG, OE, and IB initiated the project. SLB and MG analyzed and interpreted the bubble grade data. All authors conducted the experimental work and contributed to writing and revising the manuscript.

## Conflict of Interest Statement

SLB was employed by SLB Consulting. The remaining authors declare that the research was conducted in the absence of any commercial or financial relationships that could be construed as a potential conflict of interest.
